# Seascapes and foraging success: Movement and resource discovery by a benthic marine herbivore

**DOI:** 10.1002/ece3.9243

**Published:** 2022-09-11

**Authors:** Kathleen A. MacGregor, Ladd E. Johnson

**Affiliations:** ^1^ Département de Biologie Université Laval Québec Québec Canada; ^2^ Institut Maurice‐Lamontagne Pêches et Océans Canada Mont‐Joli Québec Canada

**Keywords:** drift kelp, foraging behavior, Gulf of Saint Lawrence, northwest Atlantic, rocky subtidal, sand substratum, *Strongylocentrotus droebachiensis*, urchin barren

## Abstract

Spatially concentrated resources result in patch‐based foraging, wherein the detection and choice of patches as well as the process of locating and exploiting resource patches involve moving through an explicit landscape composed of both resources and barriers to movement. An understanding of behavioral responses to resources and barriers is key to interpreting observed ecological patterns. We examined the process of resource discovery in the context of a heterogeneous seascape using sea urchins and drift kelp in urchin barrens as a model system. Under field conditions, we manipulated both the presence of a highly valuable resource (drift kelp) and a barrier to movement (sandy substratum) to test the interacting influence of these two factors on the process of resource discovery in barren grounds by urchins. We removed all foraging urchins (*Strongylocentrotus droebachiensis*) from replicate areas and monitored urchin recolonization and kelp consumption. We tested two hypotheses: (1) unstable substratum is a barrier to urchin movement and (2) the movement behavior of sea urchins is modified by the presence of drift kelp. Very few urchins were found on sand, sand was a permeable barrier to urchin movement, and the permeability of this barrier varied between sites. In general, partial recolonization occurred strikingly rapidly, but sand slowed the consumption of drift kelp by limiting the number of urchins. Differences in the permeability of sand barriers between sites could be driven by differences in the size structure of urchin populations, indicating size‐specific environmental effects on foraging behavior. We demonstrate the influence of patchy seascapes in modulating grazing intensity in barren grounds through modifications of foraging behavior. Behavioral processes modified by environmental barriers play an important role in determining grazing pressure, the existence of refuges for new algal recruits, and ultimately the dynamics of urchin‐algal interactions in barren grounds.

## Graphical abstract

We examined the process of resource discovery in the context of a heterogeneous seascape using sea urchins and drift kelp as a model system and modifying the seascape through the addition of sand barriers. Very few urchins were found on the sand, sand was a permeable barrier to urchin movement, and the permeability of this barrier varied between sites. Behavioural processes modified by the environment play an important role in determining grazing pressure, the existence of refuges for new algal recruits, and ultimately the dynamics of urchin‐algal interactions.

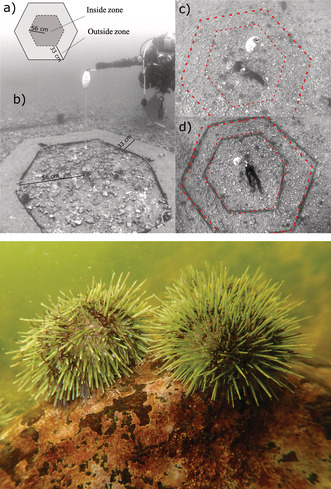

## 1 INTRODUCTION

Herbivores are important structuring forces in many biological systems, affecting not only the plants they consume but also the communities and ecosystems dependent on the primary production and habitat that plant assemblages provide (Hempson et al., 2015; Lubchenco & Gaines, 1981). The relationships between herbivores and plants are fundamental components of the functioning of biological communities and ecosystems and are central to both population biology (Holling, 1992; MacArthur & Pianka, 1966) and the study of individual behavior (Tinbergen, 1963). Linking foraging theory to the surrounding landscape is essential to develop a complete understanding of herbivore–plant interactions (Ferrario et al., 2021).

It is essential to consider the influence of the landscape through which an organism is moving when looking at individual movement behaviors. Spatially concentrated resources result in patch‐based foraging in which organisms first search for, evaluate, and then exploit concentrated areas of resources (reviewed in Stephens & Krebs, 1986). The process of locating and exploiting resource patches (how to search) and the detection and choice of patches (where to eat) both involve moving through an explicit landscape. The landscape, a mosaic of patches of habitat with characteristic features such as a dominant vegetation type, is a fundamental element of foraging. Not all landscapes or patches in a landscape will provide the same opportunities for movement or detection of both resources and threats. One way of classifying landscape patches is by characterizing the resistance they provide for movement through them (Gherghel & Papeş, 2015; Schooley & Wiens, 2004). The increase in individual movement data (e.g., technological advances in tracking methods) combined with detailed mapping (e.g., satellite imagery) has increasingly allowed detailed examinations of movement through patchy landscapes (Haynes & Cronin, 2006; Leblond et al., 2011; Singh et al., 2016; Vanbianchi et al., 2018). For example, wolves use trails and roads to facilitate their movement through a natural landscape, which has altered their interactions with prey such as caribou (James & Stuart‐Smith, 2000). Conversely, resistance to movement can be created as either physical or as behavioral impediments to moving. For example, physical impediments to movement such as thick undergrowth or deep snow may slow movement rates (Morales & Ellner, 2002) or areas of elevated predation risk may discourage movement through certain patches (Madin et al., 2011).

In marine environments, sea urchins (Phylum Echinodermata, Class Echinoidea) are widespread and abundant benthic herbivores, and the relationship between urchin and kelp populations is one of the most well‐studied herbivore/plant interactions in marine habitats (Breen & Mann, 1976; Estes & Duggins, 1995; Kitching & Ebling, 1961; Scheibling et al., 2013), due to the destructive nature of urchin grazing, which can switch kelp forests into urchin‐dominated barren grounds (Graham, 2004; Lawrence, 1975; Ling et al., 2010). Barren grounds are characterized by a lack of large fleshy macroalgae, generally high densities of urchins, and extreme resource limitation. Despite the importance of food resources in these habitats, little attention has been paid to measuring foraging behavior in the field, particularly how it varies with extrinsic (e.g., seascape, temperature, or water movement) and intrinsic (e.g., reproductive state and hunger) conditions (but see Estes & Steinberg, 1988; Suskiewicz & Johnson, 2017). Urchins living in barren grounds often depend on drift kelp as a form of ecological subsidy (Filbee‐Dexter et al., 2018; Vanderklift & Wernberg, 2008). Drift kelp is, however, an unpredictable and patchy resource, as it depends on disturbance processes that dislodge or damage kelp in adjacent or distant localities and the subsequent currents (e.g., tidal and wind‐generated) that transport and distribute them (de Bettignies et al., 2012; Krumhansl & Scheibling, 2011). Foraging behavior that confers the ability to rapidly locate and exploit these unpredictable subsidies is thus essential. Urchins, therefore, provide an ideal model system to examine the process of resource discovery and foraging behavior in subtidal seascapes.

The perceptual range of invertebrates has been demonstrated to provide useful information to foraging individuals at small spatial scales, generally through the detection of chemical cues (e.g., freshwater snails: Kawata & Agawa, 1999; and marine invertebrates: Wyeth et al., 2006). Given their small body size and limited movements (1–5 m d^−1^ for *Strongylocentrotus droebachiensis* [Müller] in the Gulf of Saint Lawrence [Dumont et al., 2006]), a “landscape” relevant for urchin foraging is on the order of meters or 10s‐of‐meters square. Past work on the behavior of urchins has identified environmental factors that influence their movement behavior, including water motion (Frey & Gagnon, 2015; Morse & Hunt, 2013), the sweeping action or abrasion of algal fronds (Gagnon et al., 2006; Konar, 2000; Konar & Estes, 2003), food availability (Harrold & Reed, 1985; Mattison et al., 1977), and intense intraspecific competition in severely food‐limited barren grounds (Narvaez et al., 2020). Although previous work has suggested that urchins can detect and move toward (or away from) stimuli such as drift algae (or predators), (e.g., Garnick, 1978; Scheibling & Hamm, 1991), more recent work has demonstrated a much more limited response of urchins to kelp stimuli and almost no response to predator stimuli in the field (Harding & Scheibling, 2015). Finally, many studies have implicitly assumed that soft substrata act as a natural barrier and can provide protection from urchin grazing (Andrew & Choat, 1985; Himmelman & Nédélec, 1990; Leinaas & Christie, 1996; Sebens, 1985). There has been, however, relatively little explicit testing of this hypothesis (but see Ferrario et al., 2021; Laur et al., 1986).

Here, we examine the process of resource discovery in the context of a heterogeneous seascape using urchins and drift kelp as a model system. In the field, we experimentally tested two hypotheses centered on the interactions between behavior and environment: (1) unstable substratum acts as a barrier to urchin resource discovery, and (2) sea urchins can perceive drift kelp, a highly valuable and scarce food resource. Specifically, we predicted that barriers of sand (a soft and unstable substratum) would deter sea urchin movement and that urchins would be able to detect drift kelp and modify their behavior, resulting in more urchins risking crossing unstable substrata in the presence of high‐quality food. As a result of the ability to detect drift kelp, we predicted that urchins would not only aggregate on drift kelp but would be attracted to the area surrounding the high‐quality food in greater numbers. We repeated the same manipulative experimental protocol at multiple sites to determine the generality of our findings.

## 2 MATERIALS AND METHODS

### 2.1 Study region and selection of sites

**FIGURE 1:**
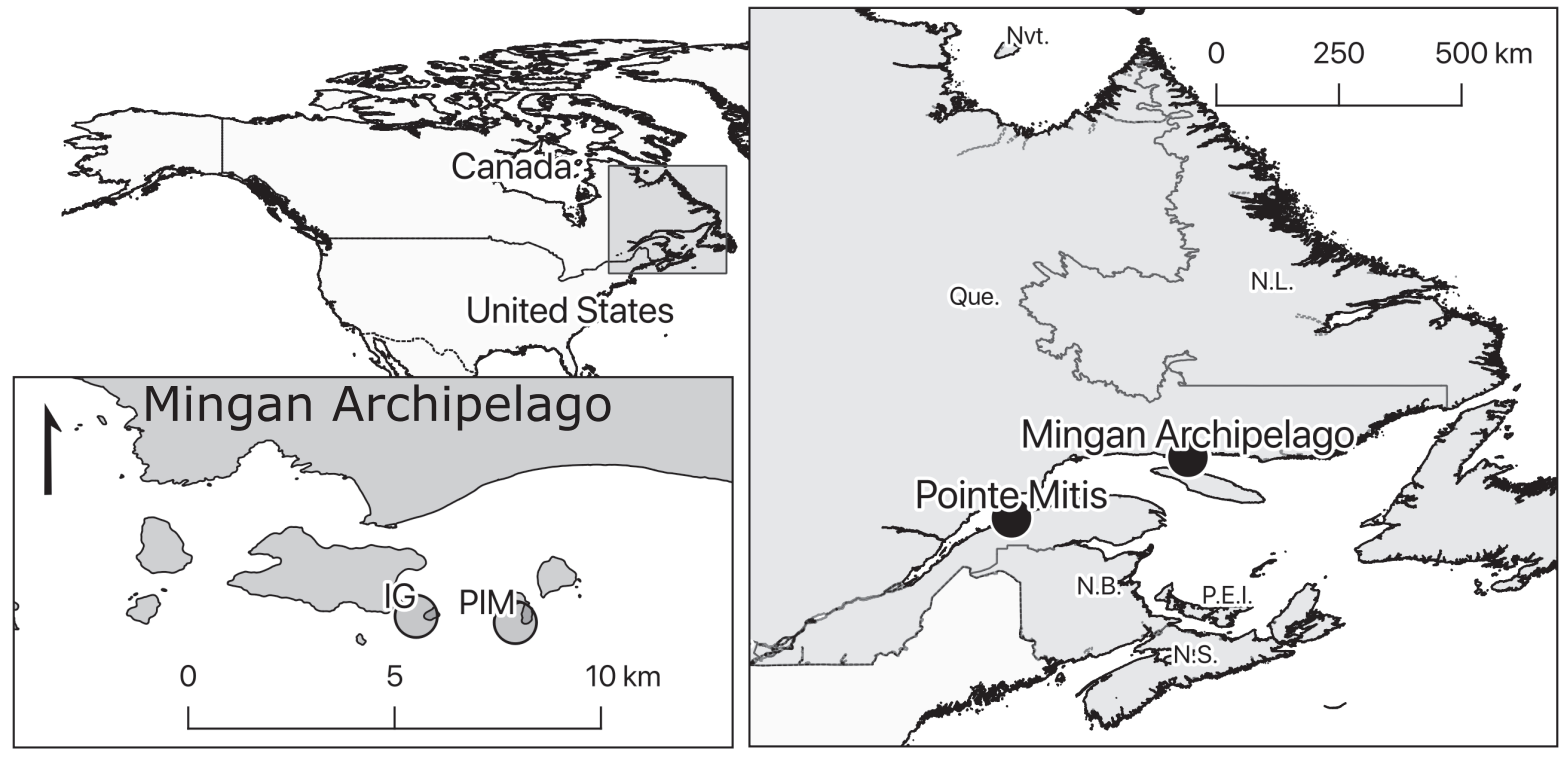
Study sites in the Gulf of Saint Lawrence (Quebec, Canada). Two sites are in the Mingan Archipelago on the north shore (Petite Île au Marteau [PIM] and Île aux Goélands [IG]) and one site on the south shore of the maritime estuary (Baie de Pointe‐Mitis [BPM]).

Fieldwork was done in the Gulf of Saint Lawrence (Quebec, Canada) where three subtidal rocky sites were selected to represent replicates of semi‐protected, 10‐m‐deep barren grounds dominated by the green sea urchin, *Strongylocentrotus droebachiensis*. Two sites were west‐facing bedrock sites in the Mingan Archipelago (50°14′N 063°36′W; Petite Île au Marteau [PIM] and Île aux Goélands [IG]), and one site was a north‐facing, cobble‐dominated site on the south shore of the Saint Lawrence maritime estuary (48°41′N 068°01′W; Baie de Pointe‐Mitis [BPM]) (Figure 1). All sites were urchin barren grounds with similar densities of large urchins (test diameter > 20 mm), a complete absence of foliose macroalgae and high cover of crustose coralline algae on all rocky substrata. All sites were predominantly rocky and presented few to no natural substratum barriers to urchin movement. Kelp (primarily *Alaria esculenta* [Linnaeus] and *Laminaria digitata* [Hudson]) were restricted to shallow water fringes (<2‐m depth) adjacent to each site. All experiments were conducted during the summer (June–August) of 2012 at 8–12 m below MLLW.

Green sea urchins demonstrate an ontogenetic switch in foraging behavior at 20‐mm test diameter; below this size, urchins are cryptic and do not actively move in search of food (Dumont et al., 2004). We therefore concentrated our manipulations on large actively foraging urchins only. Additionally, green sea urchin movement is affected by water movement (Lauzon‐Guay & Scheibling, 2007); therefore, relative water movement during manipulations was measured using clod cards (Doty, 1971) deployed during each 24‐h period of experimental manipulation (*n* = 4 per deployment). At all sites, we sampled urchin populations immediately before beginning manipulations by collecting all urchins in 0.25 m^2^ quadrats (*n* = 3–5) and recording urchin numbers and diameters later in the laboratory. Wet weight biomass was estimated from a relationship between test diameter and wet weight established during concurrent fieldwork at these same three sites (Appendix S1: Estimation of biomass).

### 2.2 Unstable substrata: A barrier to urchin movement?

We tested for the interacting effects of the presence of drift kelp, a valuable but spatially and temporally unpredictable resource, and the nature of the surrounding substratum on urchin behavior using in situ urchin clearings. Although the most detailed information on behavior is obtained by following individuals through time (Turchin, 1998), such observations are difficult underwater (but see Dumont et al., 2007; Konar, 2000). Recolonization of areas where urchins have been removed (i.e., clearings) thus provides an alternative experimental approach for inferring movements by recording changes in density through time. Clearings have many benefits, not least that they allow quicker and easier manipulation and replication of experimental factors.

**FIGURE 2:**
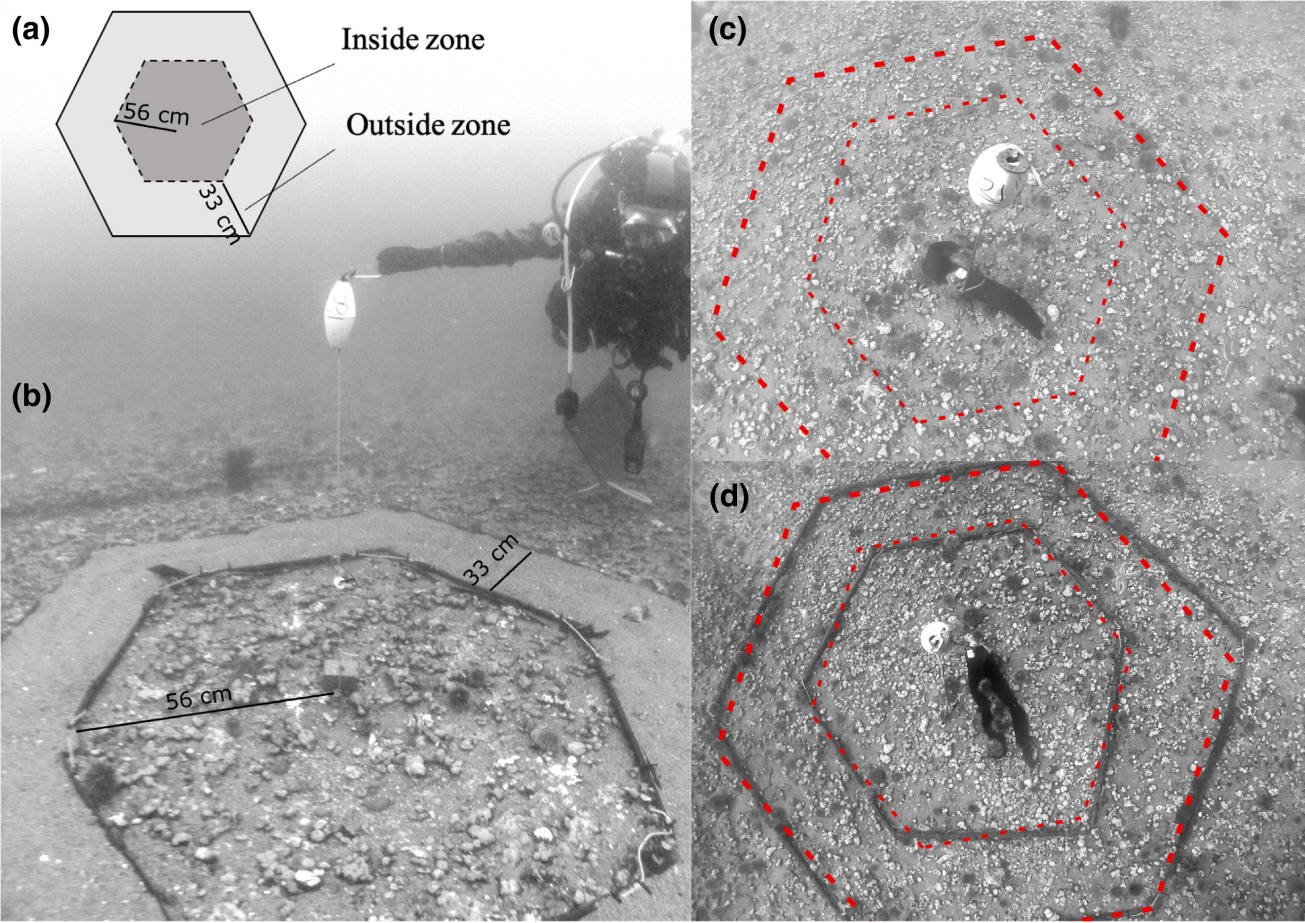
Experimental setup for in situ subtidal manipulations. (a) Schematic representation of the manipulated areas showing both the inside and outside zones; (b) Sand‐substratum treatment with central marker and subsurface float; (c) Control‐substratum treatment with kelp; (d) Rebar procedural‐control treatment with kelp.

We performed a fully crossed factorial experiment in which we removed all large urchins (test diameter > 20 mm), manipulated both the presence of drift kelp and the composition of the surrounding substratum and then monitored subsequent recolonization by urchins. We replicated this experiment at two of our three sites (IG and PIM on June 23 and July 19, respectively). At each site, large urchins were removed from 18 circular areas (radius of 89 cm; 2.5 m^2^), which were separated by at least 3 m (outer edge to outer edge) and identified with markers consisting of subsurface floats (Figure 2). We monitored movement back into these areas at 12‐, 24‐, and 48‐h intervals after urchin removal by counting the large urchins in both the central area (“inside zone”) and surrounding area (“outside zone”). For treatments with added kelp, the number of urchins in direct contact with the piece of kelp was also recorded. Kelp (added or not) and substratum treatments (three levels) were applied in a fully crossed factorial design. The presence of drift kelp was manipulated by attaching a 50‐g piece cut from the blade of freshly collected kelp (*Laminaria digitata* Hudson) to the central marker in the inside zone of half the units. Three levels of substratum treatments were applied in the outside zone: (1) sand freshly collected from a nearby intertidal beach, installed in a band 33‐cm wide to a depth of 2–4 cm and maintained in place with a border of 19‐mm diameter steel rebar on both inner and outer edges (“Sand”); (2) a procedural control consisting of rebar alone (“Rebar”); and (3) a control with no substratum manipulation (“Control”). To measure daily consumption rates, we collected, weighed, and replaced the piece of kelp after 24 h and collected and weighed the remaining kelp after 48 h. Urchins in contact with the kelp during the 24‐h visit were gently removed and left in place; most remained in contact with the newly installed piece of kelp.

### 2.3 Attraction or retention: Are sea urchins able to perceive drift kelp?

We performed a complementary second field experiment to examine the role attraction plays in the process of resource discovery by urchins. Specifically, we hypothesized that attraction toward kelp pieces would result in increased numbers of urchins in the outside zone of cleared areas containing kelp, as individuals from surrounding areas moved toward this valuable resource. To do so, we removed large urchins from 10 replicate areas as described in Section 2.2 at all three sites (PIM, IG, and BPM on August 11, August 14, and July 26, respectively) and placed pieces of kelp in half of the clearings (as in Figure 2c). We assessed attraction to drift kelp by counting large urchins in the inside and outside zones at 3, 8, 15, 24, and 48 h after removing urchins, as well as those directly in contact with the piece of kelp after 24 and 48 h. To measure daily consumption rates, we again collected, weighed, and replaced the piece of kelp after 24 h and collected and weighed the remaining kelp after 48 h.

### 2.4 Statistical analyses

#### 2.4.1 Barriers to movement: experiment 1

The interacting effects of a substratum barrier and the presence of drift kelp on urchin recolonization were analyzed using only the 48‐h measures from the first experiment. After graphical validation of the mean–variance relationship of the data, a negative binomial distribution was selected as the best fit for the data as variances increased markedly with increasing means. Two negative binomial regressions were fit to the data for the inside zone and the outside zone, respectively, using the glm.nb function from the MASS package (R Core Team, 2020; Ripley et al., 2022) in R (Equation 1):

(1) Urchin density∼Site.exp+Kelp+Substratum+Kelp:Substratum

All factors were treated as fixed factors, since two levels for Site did not allow accurate estimation of a random factor. Model fits and assumptions were verified visually using residual plots and qqplots and by calculating variance inflation factors (Appendix S1: Barriers to movement). The significance of fixed effects was assessed by sequential deletion from the maximal model using maximum likelihood parameter estimation. Deviance change between models with and without individual terms was tested using chi‐squared tests (analysis of deviance) (Zuur et al., 2007).

#### 2.4.2 Attraction or retention: experiments 1 and 2

To separate the effects of attraction versus retention of urchins by drift kelp, all replicates with no manipulation of substratum were used; this included all of experiment 2 and also the Control replicates from experiment 1. Two generalized additive models (GAM) were fit to this data (one for the inside and one for the outside zone), using the gam function from the mgcv package (Wood, 2022) in R (Equation 2), the negative binomial distribution for the generalized linear model (GLM) portion, and including a smoothing spline through time as a function of kelp presence or absence. In addition, a blocking factor for unique site‐experiment combinations was included (Site.exp).

(2) $$\text{Urchin density in zone}∼\text{Site.exp}+\text{Kelp}+\text{spline}\left(\text{Time, by}=\text{Kelp}\right)$$

By comparing the results of kelp versus no kelp and the patterns through time in the inner vs. outer zones, we describe the attraction and retention of urchins by drift kelp. All factors were treated as fixed factors, and model fits were verified visually using residual plots, qqplots (GLM portion of the model), and plots of estimated splines (Appendix S1: Attraction/retention). The significance of parametric parameters and splines was assessed using analyses of deviance (chi‐square tests).

#### 2.4.3 Consumption of drift kelp: experiments 1 and 2

To describe how consumption of drift kelp was affected by the numbers of urchins actively grazing and the substratum barriers installed, all kelp consumption data from the 24‐ and 48‐h visits from both experiments (barrier experiment and attraction/retention experiment) were analyzed together. Including the replicates from the attraction/retention experiment increased the number of Control replicates (as no substratum manipulation was carried out during experiment 2) and, therefore, allowed a more detailed evaluation of the role of number of urchins on consumption. Experimental units done at the same site and time were considered as a block (Site.exp). The proportion of available biomass consumed in 24 h was calculated for each deployment, but all values greater than 0.8 were removed from the analysis because the number of urchins in contact with the kelp began decreasing after this threshold, presumably due to urchins leaving a depleted resource patch. The distribution of the data was best described by a beta distribution (bounded proportional data), so we used the beta function from the betareg package (Zeileis et al., 2021) in R (Equation 3).

(3) Proportion∼Site.exp+Urchins on kelp+Substratum+Urchins:Substratum

Model fit was verified visually using residual plots and qqplots and by calculating variance inflation factors (Appendix S1: Consumption). The significance of parameters was assessed using analysis of deviance (Type II chi‐square tests for unbalanced sample sizes).

We then tested whether the mean proportion of kelp consumed (Control replicates only) across both experiments could be explained by relative water movement (clod card loss). Replicates of kelp consumption were averaged within each 24‐h period of each experimental deployment to give a single estimate of consumption per 24‐h period, which was matched with 24‐h relative water movement for the analysis. We fit a beta regression (bounded proportional data) using the beta function from the betareg package (Equation 4):

(4) Mean proportion consumed∼Relative water movement

Model fit was verified visually using residual plots and qqplots (Appendix S1: Consumption). Significance was assessed using an analysis of deviance with a chi‐square test. We also examined the size–frequency distribution of urchin populations at the three sites and calculated density and biomass per m^2^ for large urchins and for the total urchin population.

## 3 RESULTS

### 3.1 Unstable substrata are a permeable barrier to urchin movement

**FIGURE 3:**
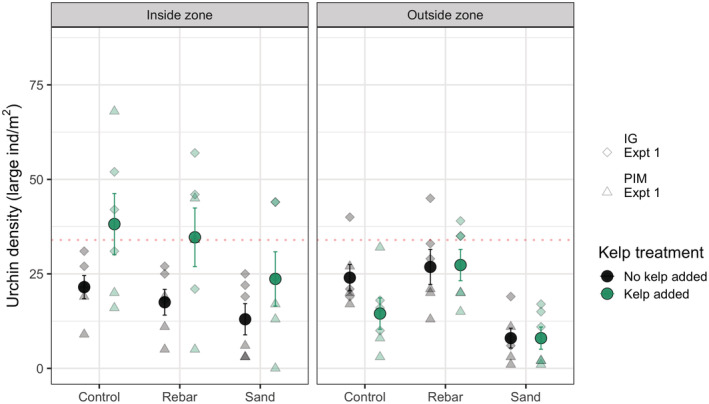
Barriers to movement: The number of large urchins in both the inside and outside zones after 48 h. Large points are means ± 1 standard error, and individual data points are shown as smaller and paler points (*n* = 3 per treatment at each of the two sites which are indicated by different shapes). The dashed red line in each panel indicates the overall mean pre‐manipulation large urchin density (see Table 4 for more detail).

Urchin density in the inside zone of clearings was higher when drift kelp was present (with most/many urchins aggregated on the piece of kelp), and substratum acted as a permeable barrier to recolonization of cleared areas (Table 1). In contrast, there was an effect of substratum but not of kelp on urchin density in the outside zone with lower densities of urchins on the Sand treatment (Table 1 and Figure 3). There was no evidence of a significant interaction between the presence of drift kelp and manipulated substratum for either the inside or the outside zone (Table 1 and Figure 3).

**TABLE 1:** Results of analysis of barriers to movement: Analysis of deviance table (likelihood ratio tests of negative binomial models) is presented with beta‐coefficients (effect sizes) and 95% confidence intervals, back‐transformed to facilitate interpretation

	exp(ß)	95% CI lwr	95% CI upr	*df*	LR *χ* ^2^	Pr (>*χ* ^2^)
Inside zone						
Site (ref: IG)	0.48	0.34	0.68	1	13.92	**<0.001**
Kelp (ref: no Kelp)	1.86	1.04	3.35	3	11.77	**<0.001**
Substratum (ref: Control)				4	8.03	**0.02**
Rebar	0.79	0.43	1.43			
Sand	0.53	0.28	0.97			
Kelp:Substratum				2	0.09	0.96
Kelp:Rebar	1.12	0.49	2.56			
Kelp:Sand	1.00	0.43	2.32			
Outside zone						
Site (ref: IG)	0.53	0.39	0.71	1	13.90	**<0.001**
Kelp (ref: no Kelp)	0.61	0.38	1.00	3	1.37	0.24
Substratum (ref: Control)				4	27.61	**<0.001**
Rebar	1.06	0.66	1.70			
Sand	0.31	0.18	0.53			
Kelp:Substratum				2	2.27	0.32
Kelp:Rebar	1.66	0.85	3.25			
Kelp:Sand	1.53	0.71	3.25			

*Note*: Coefficients correspond to the change in odds as compared to the reference level such that a coefficient of 1.78 represents a 78% increase and a coefficient of 0.25 represents a 25% decrease. In this case, confidence intervals that do not overlap with 1 indicate a significant effect. Significant effects are indicated in bold.

### 3.2 Retention and not attraction explain increased urchin densities

**FIGURE 4:**
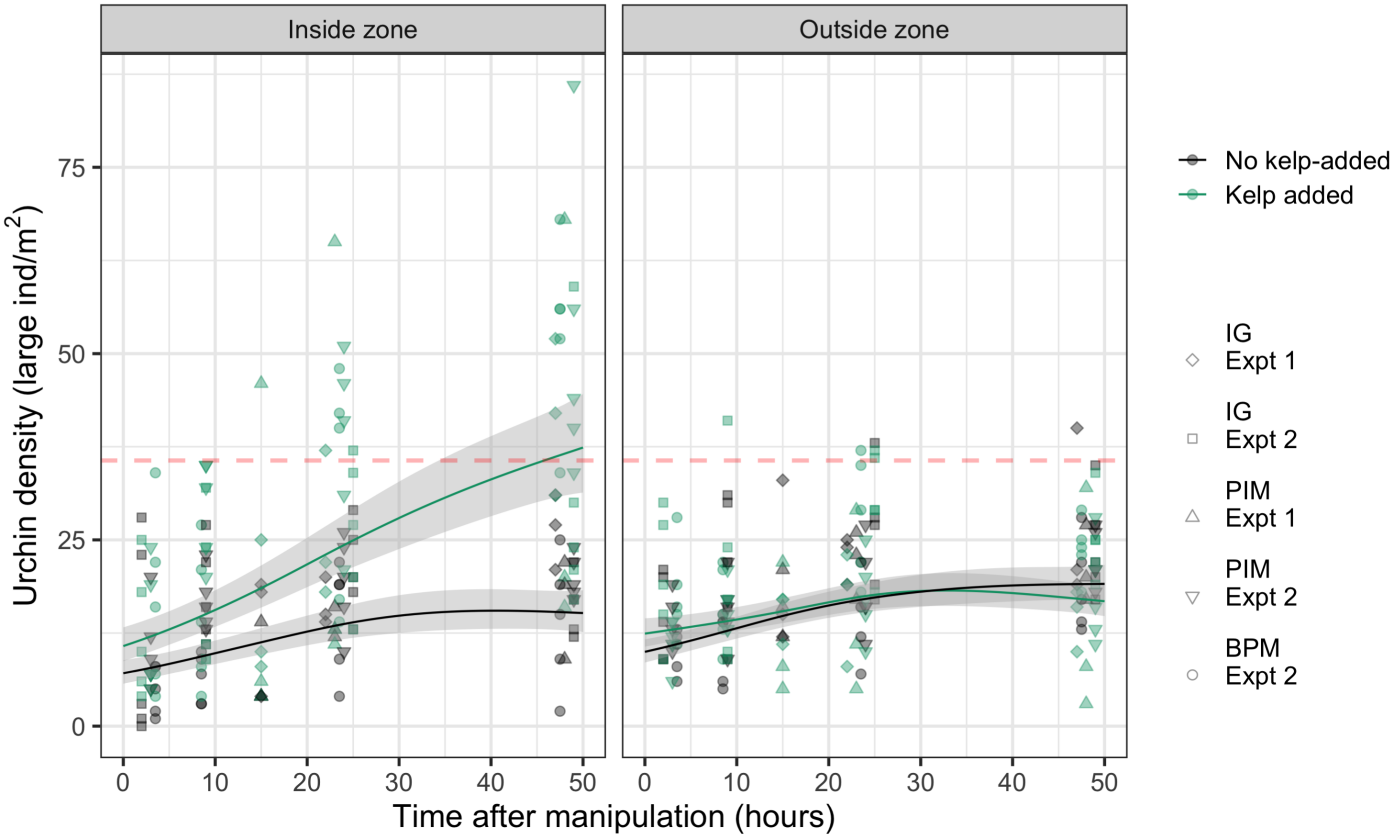
Temporal and spatial patterns of urchin aggregation on kelp. Small points are data from individual experimental units. Lines are splines of large urchin densities through time ± 1 standard error. The dashed line in each panel indicates the overall mean density of large urchins across all sites.

Retention of urchins was clearly shown by the increasing numbers of urchins found in the inside zone containing the piece of drift kelp (Table 2 and Figure 4, left panel). However, drift kelp does not appear to attract urchins, as there were no differences in the densities in the outside zone (separated from the piece of drift kelp by only 56–89 cm) between treatments throughout the experimental period (Table 2 and Figure 4, right panel). In addition, increases in urchin density through time in the outside zone were not different from those seen in the inside zone in the absence of kelp, indicating that urchins are recolonizing areas at a base rate, and only the presence of kelp (and not proximity to kelp) causes an aggregation of urchins. Interestingly, there was a highly significant effect of Site.experiment in the outside zone, but not in the inside zone, indicating that the response to kelp (i.e., only retention) is consistent across sites and times, but foraging movements can vary spatially and temporally.

**TABLE 2:** Retention and not attraction: Analysis of deviance table

	*df*	*χ* ^2^	Pr (>*χ* ^2^)
Inside zone			
*Parametric terms*			
Site.experiment	4	8.64	0.07
Kelp	1	43.86	**<0.0001**
*Approximate significance of smooth terms*			
No kelp through time		15.52	**0.001**
Kelp through time		44.27	**<0.0001**
Outside zone			
*Parametric terms*			
Site.experiment	4	20.36	**0.0004**
Kelp	1	0.255	0.61
*Approximate significance of smooth terms*			
No kelp through time		22.98	**<0.0001**
Kelp through time		8.28	**0.03**

*Note*: Significant effects are indicated in bold.

In both experiments, urchin densities increased after removal (smoothing terms were significant; Table 2). The initial increase was extremely rapid, with 25%–50% of pre‐manipulation densities typically found at the first visit 3 h after clearing (Figure 4). However, except for the kelp‐addition treatment, densities remained generally lower than pre‐manipulation densities even 48 h later (Figures 3 and 4).

### 3.3 Consumption of drift kelp

**FIGURE 5:**
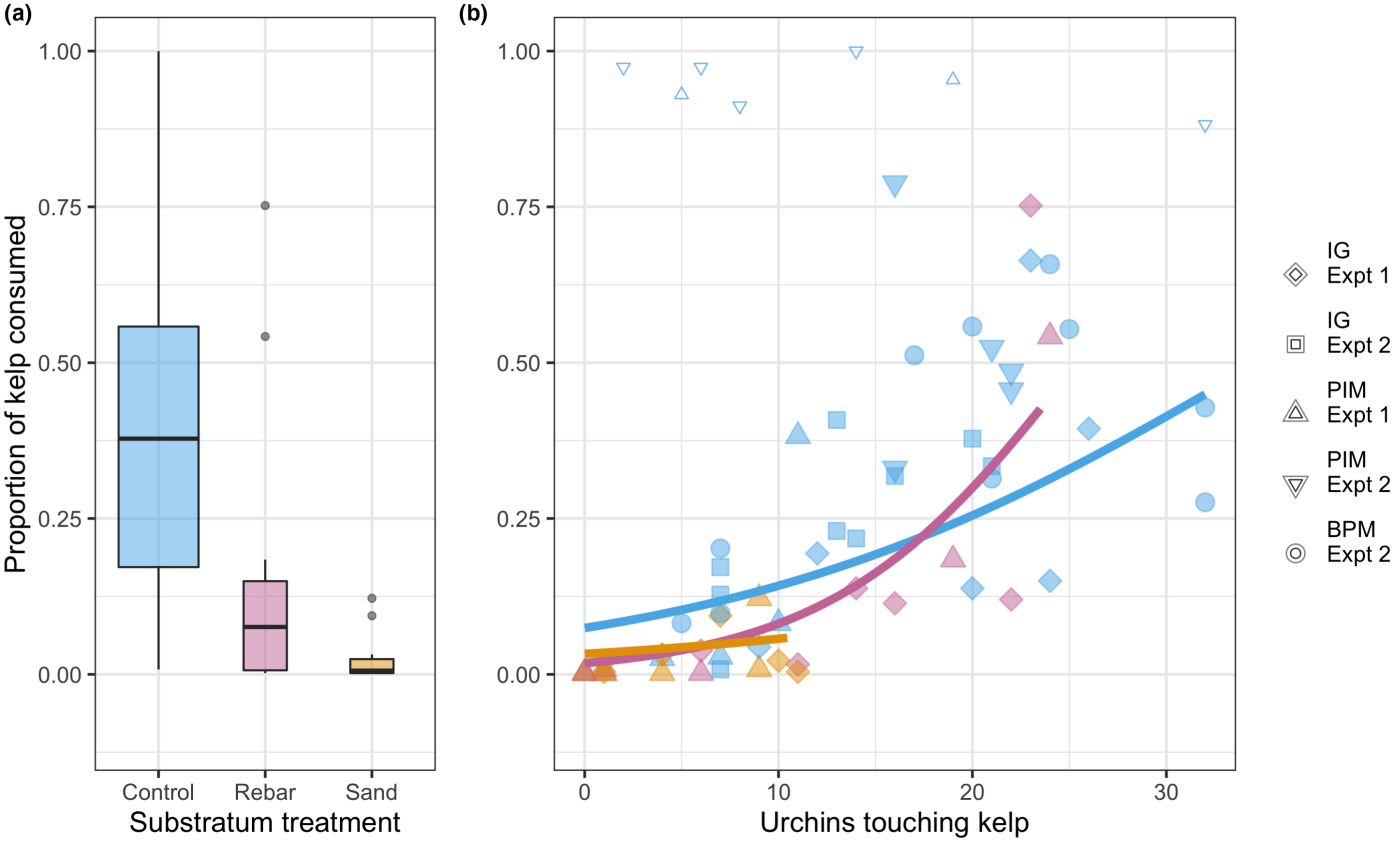
Proportion of kelp consumed per 24 h in all manipulations (barriers experiment and attraction/retention experiment combined). (a) Boxplots showing the distribution of values per substratum treatment; box widths are proportional to the square roots of the number of replicates (Sand *n* = 12, Procedural Control *n* = 12 and Control *n* = 34). (b) Proportion of kelp consumed as a function of the number of urchins in contact with the kelp. Points are colored by substratum treatment, and shapes indicate site‐experiment blocks. Unfilled points with values >0.8 were not included in the model, and lines show predicted values.

The proportion of kelp consumed increased with the number of urchins found on the kelp, but a significant interaction with substratum indicated that the number of urchins on the kelp was affected by substratum barriers (Table 3 and Figure 5). In all cases, Site.experiment had a significant effect on the response observed, indicating significant spatial and/or temporal variation in the responses observed.

**TABLE 3:** Results of the analysis of kelp consumption: Analysis of deviance table (Type II tests for unbalanced sample sizes) is presented with beta‐coefficients (effect sizes) and 95% confidence intervals, back‐transformed to facilitate interpretation

	exp(ß)	95% CI lwr	95% CI upr	*df*	*χ* ^2^	Pr (>*χ* ^2^)
Site.experiment (ref: IG expt 1)				4	10.41	**0.03**
IG expt 2	1.38	0.70	2.73			
PIM expt 1	0.95	0.53	1.71			
PIM expt 2	3.16	1.52	6.58			
BPM expt 2	1.68	0.89	3.19			
Urchins	1.08	1.04	1.11	1	38.34	**<0.0001**
Substratum (ref: Control)				2	2.94	0.23
Rebar	0.23	0.07	0.73			
Sand	0.42	0.14	1.28			
Urchins:Substratum				2	7.29	**0.03**
Urchins:Rebar	1.09	1.02	1.16			
Urchins:Sand	0.99	0.86	1.13			

*Note*: Coefficients correspond to the change in odds as compared to the reference level such that a coefficient of 1.78 represents a 78% increase and a coefficient of 0.25 represents a 25% decrease. In this case, confidence intervals that do not overlap with 1 indicate a significant effect. Significant effects are indicated in bold.

**FIGURE 6:**
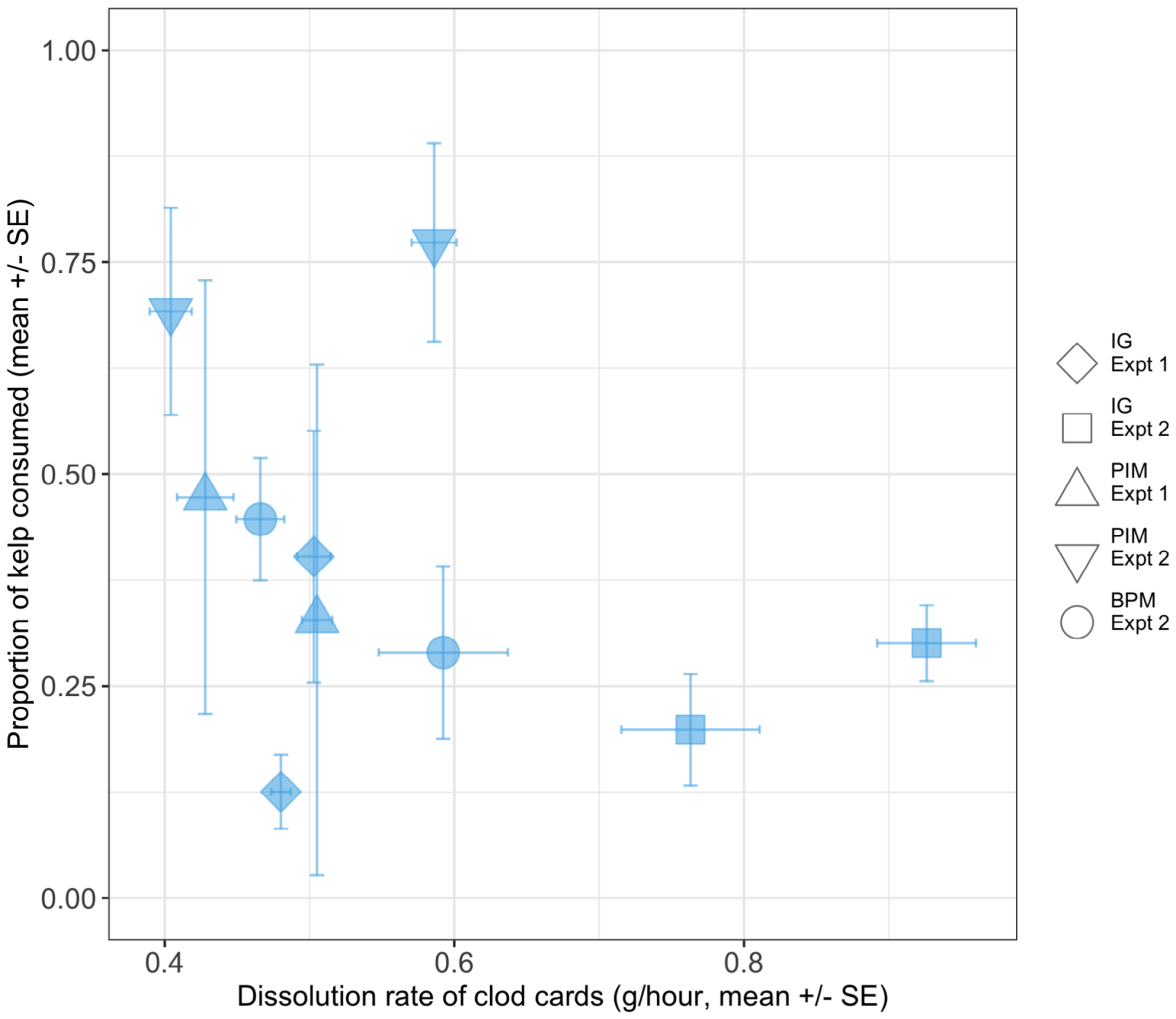
No evidence for a relationship between relative water movement (dissolution rates of clod cards) and consumption of kelp during the study period.

**FIGURE 7:**
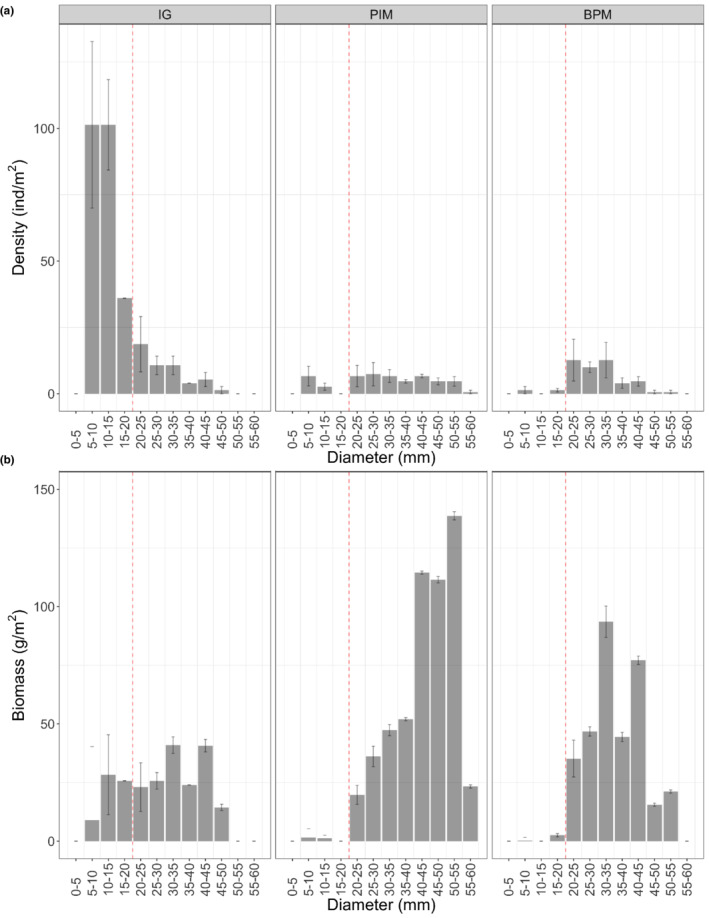
Size and biomass distribution for the three experimental sites. (a) Size distributions are shown as urchins.m^−2^ within 5‐mm bins of test. (b) Biomass distribution within the same 5‐mm bins of test diameter. The red dashed line indicates the limit defined as large actively foraging urchins in the present study (test diameter > 20 mm).

The mean proportion of kelp consumed in clearings with no substratum manipulation was not significantly related to the measures of relative water movement (dissolution rates ranged fivefold from 0.2 to 1 g h^−1^; Figure 6), indicating that differences between sites in observed recolonization and consumption rates were not related to differences in water movement we measured (χ^2^ = 1.01, *p* = .31). Site.experiment differences throughout manipulations were more marked in the outside and non‐kelp treatments, indicating that although the response to the presence of drift kelp is generalizable across sites and times, there are clearly important extrinsic factors influencing urchin foraging behavior. Although sites were chosen to have similar densities of large urchins, there were clear differences between sites in total density, total biomass and, therefore, in the proportion of total biomass at the site accounted for by large urchins (Table 4 and Figure 7).

**TABLE 4:** Summary densities and biomass per m^2^ highlighting different proportions of large to small urchins

Site	Large urchin density (ind > 20 mm/m^2^)	Total density (ind/m^2^)	Total biomass (g/m^2^)	Large urchin biomass (grams > 20 mm/m^2^)	Proportion of density accounted for by large urchins	Proportion of biomass accounted for by large urchins
IG	32 ± 5	289 ± 42	928 ± 85	583 ± 66	0.11	0.63
PIM	35 ± 7	51 ± 7	1092 ± 173	1047 ± 156	0.69	0.96
BPM	33 ± 12	48 ± 20	673 ± 276	597 ± 234	0.69	0.89

*Note*: Values presented are means ±1 standard error. Sites were selected based on having similar large urchin densities (as seen in the first column) but varied in the proportion of estimated total biomass at the site accounted for by large urchins.

## 4 DISCUSSION

### 4.1 Unstable substrata function as variably permeable barriers to urchin foraging movements

Foraging behavior plays an important role in modifying the grazing pressure in heterogeneous seascapes. Sand is often evoked as an important barrier to urchin movement (e.g., Jones & Kain, 1967) as moving across unstable substrata may increase the risk of dislodgment by predators or water motion (Laur et al., 1986). Our results support this idea with lower densities and consumption rates in areas protected by a sand barrier but also show that, in this habitat at this spatial scale, sand acts only as a permeable barrier. Encountering sand likely discourages urchins from moving forward, but if they do, it does not appear to slow them down as urchins were less commonly found on sand, an observation noted elsewhere (Leinaas & Christie, 1996; Sivertsen, 1997; Sivertsen & Hopkins, 1995) but rarely well documented (but see Ferrario et al., 2021). Minimizing time on an unstable substratum would minimize the risk of dislodgment, but the precise risks to urchins in this system are unclear. Although potential predators (decapods, sea stars, and fish) exist, they are not generally considered to exert significant predation pressure on large actively foraging urchins (Scheibling, 1996). Moreover, the Gulf of Saint Lawrence notably lacks abundant predators both currently and historically (Johnson et al., 2019). The actual risk of moving over unstable substrata in this region may, therefore, depend more on water motion than predation although we saw no effect of this factor at the levels of water motion measured at our sites.

Interestingly, the permeability of the barrier was spatially variable, with urchins at PIM being more deterred than those at IG. While this may be due to temporal differences, the two trials were done within a month of each other so seasonal differences were not at play. Instead, we attribute this finding to subtle differences in the size–frequency distribution of urchins between the two sites. Although densities of large urchins were comparable at the two sites, almost 50% of the large urchins at PIM had test diameters greater than 40 mm, whereas only about 10% of those at IG were larger than 40 mm, and mostly less than 45 mm. This difference suggests an alteration of foraging behavior at 40–45 mm test diameter, when urchins may become more dependent on solid substrata and more hesitant to cross barriers of unstable substrata, especially as drag and lift forces generally increase with increasing size (Denny et al., 1985).

### 4.2 Incentives for risk taking

Although examples of incentives inducing risky behavior in urchins have previously been documented (e.g., feeding aggregations in the presence of lobsters; Vadas et al., 1986), the ability to weigh costs and benefits requires an ability to perceive drift kelp at a relevant distance. However, the close proximity of kelp appeared to have no effect on the rate of recolonization of the outside zone in our first experiment. We attribute this lack of a response to an inability of urchins to detect kelp under field conditions unless in direct contact or very close proximity. While several lab and field studies suggest that urchins have the ability to detect chemicals emitted by predators and food (Bernstein et al., 1981; Harding & Scheibling, 2015; Scheibling & Hamm, 1991), the spatial scales over which this ability is useful in field conditions remain unclear. Indeed, even under laboratory conditions, the ability of urchins to locate food resources has sometimes been shown to be limited to far less than a meter (Klinger & Lawrence, 1985). Our second experiment supports the conclusion that detection of chemical cues by urchins under field conditions is limited to very short distances, as urchin densities only increased in the immediate vicinity of drift kelp and principally due to retention after contact. Thus, the large aggregations commonly observed on kelp (Feehan et al., 2012; Vadas et al., 1986) occur through retention of urchins that stop moving once they encounter the drift kelp and not through attraction on a larger spatial scale. This conclusion strongly suggests that although urchins are voracious herbivores that consume kelp at impressive rates (Suskiewicz & Johnson, 2017), their ability to directionally locate the source of a chemical cue in turbulent field conditions is limited.

### 4.3 Urchin foraging in barren grounds: Widely ranging or sit‐and‐wait?

Locating resources in a patchy landscape is critical for survival and will depend on the perceptual range of an animal (i.e., “from what distance can animal x detect landscape element y” [Lima & Zollner, 1996]). A large perceptual range allows detection of and directed movement toward resource patches, where an extremely restricted perceptual range, such as appears to be the case for urchins, will result in foraging that is a blind scramble through the landscape with the identification of a resource patch happening only when the patch is physically encountered. The rapid initial increase in densities in our experimental clearings suggests urchins are continually moving, but randomly distributing themselves across the seascape (Dumont et al., 2007). However, the lack of a continued increase over time is striking, especially as net displacements of 1–5 m.d^−1^ have previously been observed in this system (Dumont et al., 2006). This observation could be explained by sympatric coexistence of divergent foraging strategies in this population: “sit and wait” and “widely ranging” strategies (Evans & O'Brien, 1986; Huey & Pianka, 1981; O'Brien et al., 1990). Green sea urchins have been hypothesized to have two modes of feeding behavior relevant to foraging in barren grounds: (1) passive detritivory and (2) dispersed browsing (Mann 1985 as cited in Scheibling et al., 2020), corresponding to “sit and wait” and “widely ranging” strategies, respectively. However, the switch between these two types of foraging has mainly been attributed to site‐level contexts (quantity of drift available or topographic refuges). The existence of two types or groups of individuals at the same site displaying these two contrasting strategies or by temporal switches between these two modes of behavior by individuals could produce the observed rapid, but only partial, recolonization of the areas where we removed urchins. Differences in hunger state, recent foraging history or reproductive state are interesting potential drivers of these two divergent foraging strategies and provide ground for future explorations. Regardless, in comparison with other plant–herbivore systems, especially terrestrial ones, the “mobile” nature of drift kelp, the resource in question here, allows both strategies to work as currents can deliver the resource directly to the consumer.

## 5 CONCLUSIONS AND DIRECTIONS FOR FUTURE RESEARCH

These results demonstrate the importance of considering the landscape patchiness as well as drivers of individual variability in behavior when considering foraging strategies. Applying patch‐based foraging theories to invertebrates in marine environments requires carefully testing both perception and movement abilities of organisms in situ. Future work should scale these investigations up to larger areas (i.e., larger landscape extents) and longer timescales (e.g., Ferrario et al., 2021) Work on heterogenous submarine landscapes at these larger scales is essential for understanding their importance for ecosystem processes and linking foraging behavior to the landscape ecology of benthic habitats. For example, kelp beds have recently been suggested to be an important source of exported carbon both as subsidies to other systems (Krumhansl & Scheibling, 2011) and as a means of carbon storage (Krause‐Jensen & Duarte, 2016), but evaluating the importance of this pathway of carbon storage will require a detailed understanding of the environmental processes controlling the relationship between kelp and urchins, their most important grazer. As shown here, the role of behavior can clearly not be ignored.

## AUTHOR CONTRIBUTIONS

**Kathleen A. MacGregor:** Conceptualization (equal); data curation (lead); formal analysis (lead); methodology (equal); validation (equal); visualization (equal); writing – original draft (lead); writing – review and editing (lead). **Ladd Johnson:** Conceptualization (equal); funding acquisition (lead); methodology (equal); project administration (lead); resources (lead); supervision (lead); validation (supporting); writing – review and editing (equal).

## CONFLICT OF INTEREST

No potential conflict of interest was reported by the authors.

### OPEN RESEARCH BADGES

This article has earned an Open Data badge for making publicly available the digitally‐shareable data necessary to reproduce the reported results. The data is available at https://doi.org/10.5061/dryad.2z34tmppz.

## Supporting information


Appendix S1

## Data Availability

All data used in this article are available on Dryad (https://doi.org/10.5061/dryad.2z34tmppz).
