# Supplementary material for: Seascapes and foraging success: Movement and resource discovery by a benthic marine herbivore
Source: Ecol Evol. 2022 Sep 11;12(9):e9243. doi: 10.1002/ece3.9243 (PMC9465196; doi:10.1002/ece3.9243)
Supplement: Supplementary file 1 — Appendix S1 [file ECE3-12-e9243-s001.pdf]

Supplementary Material

Seascapes and foraging success: movement and resource discovery by a benthic marine herbivore

Kathleen A. MacGregor[1,2*] and Ladd E. Johnson[1]

[1]Université Laval, Département de biologie, Québec, Québec G1V 0A6, Canada
Present address: [2]Institut Maurice-Lamontagne, Pêches et Océans Canada, Mont-Joli, Québec G5H 3Z4, Canada

1. Estimation of biomass

Wet weight biomass of urchins for which only diameter was measured in the field was estimated from a relationship between test diameter and wet weight established during concurrent fieldwork at these same three sites where both test diameter and wet weight were samples for all size classes.

A polynomial regression (with the form indicated by 'form.full') was fit to all test diameter data collected from our field sites (model = 'M.biomass'). This relationship was highly significant, and the model described 97% of the variability in the data. Model fit was evaluated graphically and apart from several outlier or extreme values, assumptions of homogeneous variances and normality were met. This was considered reliable enough to estimate wet weight biomass for our sampled urchins by using the regression equation as a function ('biom.function': Fig. S1).

```
> form.full <- Wet_wt ~ poly(Diam, 3, raw = TRUE)
> M.biomass <- lm(form.full, data=Size[!is.na(Size$Wet_wt),])

> summary(M.biomass)


Coefficients:
                             Estimate Std. Error t value Pr(>|t|)
(Intercept)                -4.177e-01  4.315e-01  -0.968    0.333
poly(Diam, 3, raw = TRUE)1  6.332e-02  6.668e-02   0.950    0.343
poly(Diam, 3, raw = TRUE)2  1.078e-04  2.808e-03   0.038    0.969
poly(Diam, 3, raw = TRUE)3  3.977e-04  3.453e-05  11.516   <2e-16 ***
---
Signif. codes:  0 '***' 0.001 '**' 0.01 '*' 0.05 '.' 0.1 ' ' 1

Residual standard error: 2.001 on 1019 degrees of freedom

Multiple R-squared:  0.9677,  Adjusted R-squared:  0.9676

F-statistic: 1.018e+04 on 3 and 1019 DF,  p-value: < 2.2e-16



> biom.function <- function(x) {
  -0.4177 + 0.0633*x + 0.0001*x^2 + 0.0004*x^3
```

}

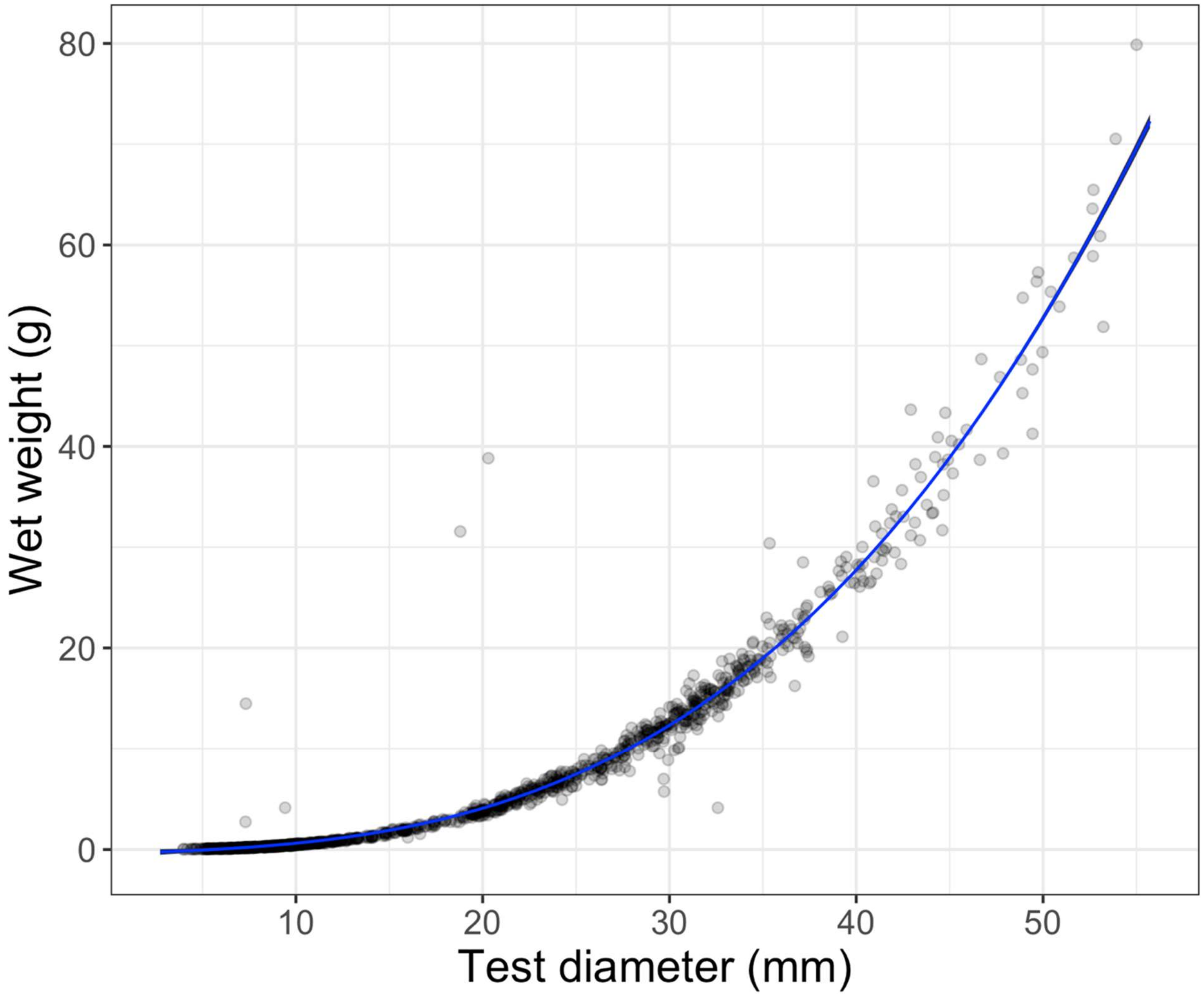

Figure S1. Relationship between test diameter and wet weight for urchins at our three field sites. All data points are for individual urchins and the blue line is predicted values from the fit regression (± 1 standard error, although the error is so small that this is barely visible). The regression equation representing this line (biom.function from above) was used to estimate wet weight for individual urchins of a known test diameter.

2. Barriers to movement: experiment 1

The interacting effects of a substratum barrier and the presence of drift kelp on urchin recolonization were analyzed using only the 48-hr measures from the first experiment.

First, we evaluated the mean-variance relationship of this data in order to compare the fit of poisson (mean = variance) or negative binomial (variance allowed to increase with increasing

means) distributions, both of which are appropriate for count data (Fig. S2). The negative binomial distribution clearly better represents the mean-variance relationship of our data (the poisson distribution – red line – consistently underestimates variance at higher mean values).

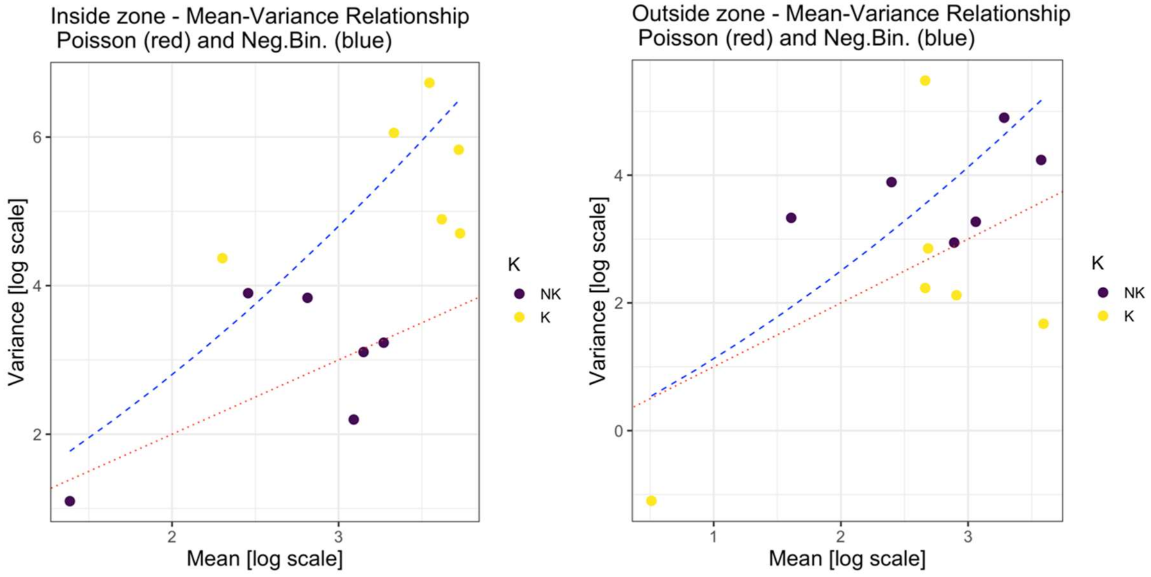

Figure S2. Mean-variance relationship of the data from experiment 1, for the inside (left panel) and outside (right panel) zones. Each point represents a treatment combination for which a mean and variance was calculated. The dotted red line follows the 1:1 line, where the mean = the variance. For the assumptions of a poisson regression to be met, the data should fall along (or be evenly distributed both above and below) this line. The dashed blue line represents a negative binomial distribution established using the means from our data set.

Two negative binomial regressions were fit to the data for the inside zone and the outside zone, respectively, using the glm.nb function from the MASS package (Ripley et al., 2022).

$$Urchin\ density \sim Site.exp + Kelp + Substratum + Kelp{:}Substratum$$

Inside zone

First, we specify the model formula and fit our negative binomial regression to the data for the inside zone:

```
form <- Den ~  fSet_Site + fK + fSub + fK:fSub
> M1.int48hr <- MASS::glm.nb(formula = form, data = M1.int48hr_data)
```

Den = Urchin density
fSet_Site = Site.experiment
fK = Kelp

fSub = Substratum

Second, we evaluated model fit and assumptions, both graphically (Fig. S3) and by calculating variance inflation factors.

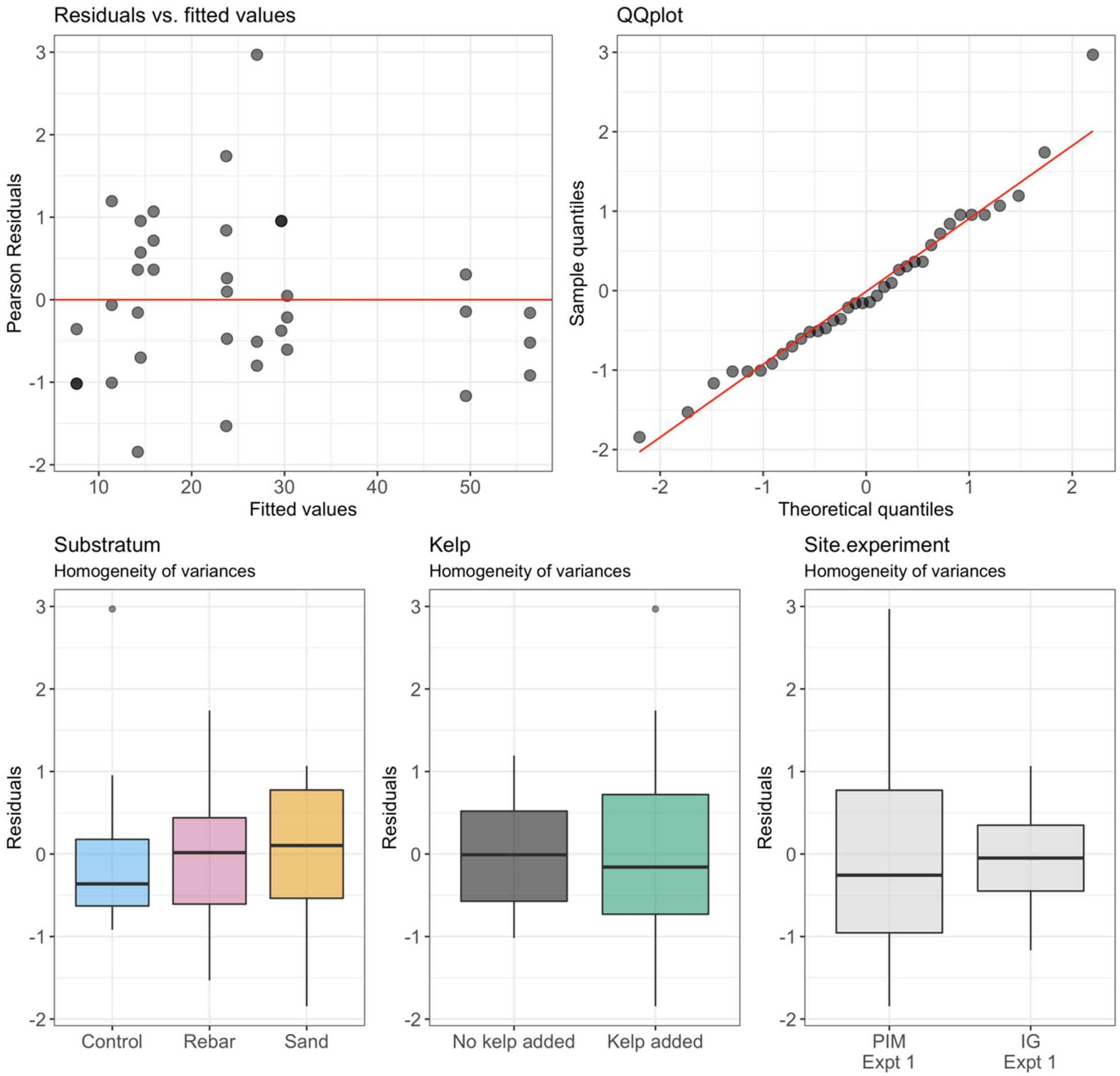

Figure S2. Visual evaluation of model fit and assumptions. The top two panels show residuals versus fitted values with no worrying patterns in the spread (left) and a quantile-quantile plot showing good fit. The bottom three plots show residuals by the three factors included in the model with no extreme heterogeneity of residuals evident.

Variance inflation factors (see below) are low (GVIF$^{\wedge(1/(2*Df)}$ > 4) in all cases, indicating no problems with the model.

```
                GVIF Df GVIF^(1/(2*Df))
fSet_Site 1.000500  1        1.000250
fK        2.855122  1        1.689711
fSub      4.516223  2        1.457786
fK:fSub   8.552732  2        1.710119
```

The significance of fixed effects was assessed by sequential deletion from the maximal model using maximum likelihood parameter estimation.

Full model formula in R notation:

```
> form <- Den ~  fSet_Site + fK + fSub + fK:fSub
```

Drop interaction term and compare to the full model:

```
                          Model   Test    2xlog-lik.    df LR stat. Pr(Chi)
1 fSet_Site + fK + fSub                   -273.51    NA       NA      NA
2 fSet_Site + fK + fSub + fK:fSub 1 vs 2  -273.43     2     0.09    0.96
```

Interaction term is not significant, so drop it from the model. Now compare a model with Substratum to the model excluding the interaction term and Substratum:

```
                  Model   Test    2 x log-lik.    df LR stat. Pr(Chi)
1        fSet_Site + fK                -281.54    NA       NA      NA
2 fSet_Site + fK + fSub 1 vs 2         -273.51     2     8.03    0.02
```

Substratum term is significant. Now compare a model with Kelp to the model excluding the interaction term and Kelp:

```
                  Model   Test    2 x log-lik.    df LR stat. Pr(Chi)
1      fSet_Site + fSub                -285.28    NA       NA      NA
2 fSet_Site + fK + fSub 1 vs 2         -273.51     1    11.77       0
```

Kelp term is significant. Now compare a model with Site to the model excluding the interaction term and Site.experiment:

```
                 Model   Test    2 x log-lik.    df LR stat. Pr(Chi)
1            fSub + fK                -287.44    NA       NA      NA
2 fSet_Site + fK + fSub 1 vs 2         -273.51     1    13.92       0
```

Site.experiment term is significant.

Outside zone

First, we specify the model formula and fit our negative binomial regression to the data for the inside zone:

```
> form <- Den ~ fSet_Site + fK + fSub + fK:fSub
```

```
> M1.out48hr <- glm.nb(formula = form, data = M1.out48hr_data)
```

Den = Urchin density
fSet_Site = Site.experiment
fK = Kelp
fSub = Substratum

Second, we evaluated model fit and assumptions, both graphically (Fig. S4) and by calculating variance inflation factors.

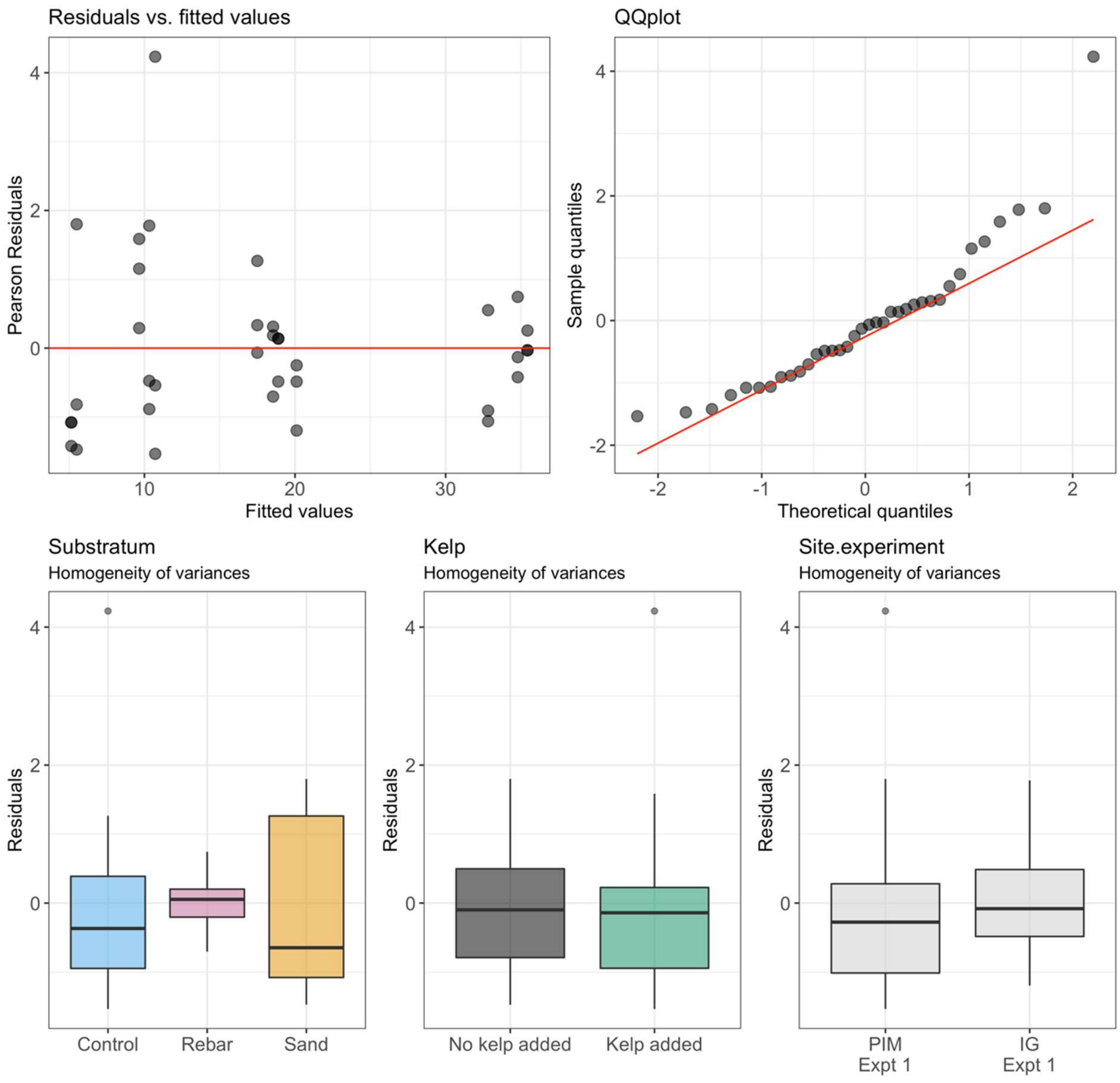

Figure S3. Visual evaluation of model fit and assumptions. The top two panels show residuals versus fitted values with no worrying patterns in the spread (left) and a quantile-quantile plot showing good fit; with a negative binomial distribution, this evaluation (of normality) is much less important, also. The bottom three plots show residuals by the three factors included in the

model with no extreme heterogeneity of residuals evident, although there is more difference here between Substratum treatments than in the Inside zone.

Variance inflation factors (see below) are low ($GVIF^{(1/(2*Df)}$ > 4) in all cases, indicating no problems with the model.

```
               GVIF Df GVIF^(1/(2*Df))
fSet_Site 1.001328  1        1.000664
fK        2.791282  1        1.670713
fSub      3.786998  2        1.394999
fK:fSub   7.491322  2        1.654397
```

The significance of fixed effects was assessed by sequential deletion from the maximal model using maximum likelihood parameter estimation.

Full model formula in R notation:

```
> form <- Den ~  fSet_Site + fK + fSub + fK:fSub
```

Den = Urchin density
fSet_Site = Site.experiment
fK = Kelp
fSub = Substratum

Drop interaction term and compare to the full model:

```
                    Model     Test    2 x log-lik.  df LR stat. Pr(Chi)
1 fSet_Site + fK + fSub                  -242.68    NA       NA      NA
2 fSet_Site + fK + fSub + fK:fSub 1vs2  -240.41     2     2.27    0.32
```

Interaction term is not significant, so drop it from the model. Now compare a model with Substratum to the model excluding the interaction term and Substratum:

```
                  Model   Test    2 x log-lik.    df LR stat. Pr(Chi)
1        fSet_Site + fK                -270.29    NA       NA      NA
2 fSet_Site + fK + fSub 1 vs 2         -242.68     2    27.61       0
```

Substratum term is significant. Now compare a model with Kelp to the model excluding the interaction term and Kelp:

```
                  Model   Test    2 x log-lik.    df LR stat. Pr(Chi)
1      fSet_Site + fSub                -244.04    NA       NA      NA
2 fSet_Site + fK + fSub 1 vs 2         -242.68     1     1.37    0.24
```

Kelp term is not significant. Now compare a model with Site to the model excluding the interaction term and Site.experiment:

```
                  Model   Test    2 x log-lik.    df LR stat. Pr(Chi)
1             fSub + fK                -256.57    NA       NA      NA
2 fSet_Site + fK + fSub 1 vs 2         -242.68     1     13.9       0
```

Site.experiment term is significant.

3. Attraction/retention : experiments 1 and 2

To separate the effects of attraction versus retention of urchins by drift kelp, all replicates with no manipulation of substratum were used; this included all of experiment 2 and also the Control replicates from experiment 1. Two generalized additive models (GAM) were fit to this data (one for the inside and one for the outside zone), using the gam function from the mgcv package (Wood, 2022), the negative binomial distribution for the generalized linear model (GLM) portion, and including a smoothing spline through time as a function of kelp presence or absence. In addition, a blocking factor for unique site-experiment combinations was included (Site.exp).

$$Urchin\ density\ in\ zone \sim Site.exp + Kelp + spline(Time, by = Kelp)$$

Model formula in R notation:

```
> form <- Den ~ fSet_Site + fK + s(TaC, by = fK)
```

Den = Urchin density
fSet_Site = Site.experiment
fK = Kelp
TaC = Time after clearing (in hours)

Inside zone

Model fit was verified visually using residual plots and qqplots (GLM portion of the model; Fig. S4) and plots of estimated splines (Fig. S5).

```
> summary(M3.int)

Family: Negative Binomial(3.849)
Link function: log

Formula:
Den ~ fSet_Site + fK + s(TaC, by = fK)

Parametric coefficients:
               Estimate Std. Error z value Pr(>|z|)
(Intercept)     2.63025    0.14191  18.535  < 2e-16 ***
fSet_SiteIG_9   0.02612    0.16223   0.161    0.872
fSet_SitePIM_4 -0.17970    0.18644  -0.964    0.335
fSet_SitePIM_8  0.20717    0.16118   1.285    0.199
fSet_SitePM_7  -0.10719    0.16222  -0.661    0.509
fKK             0.59799    0.09029   6.623 3.52e-11 ***
---
Signif. codes:  0 '***' 0.001 '**' 0.01 '*' 0.05 '.' 0.1 ' ' 1

Approximate significance of smooth terms:
              edf Ref.df Chi.sq  p-value
```

```
s(TaC):fKNK 1.893  2.219  15.52 0.000667 ***
s(TaC):fKK  1.773  2.095  44.27  < 2e-16 ***
---
Signif. codes:  0 '***' 0.001 '**' 0.01 '*' 0.05 '.' 0.1 ' ' 1

R-sq.(adj) =  0.442   Deviance explained = 42.1%
-REML = 587.81  Scale est. = 1         n = 156
```

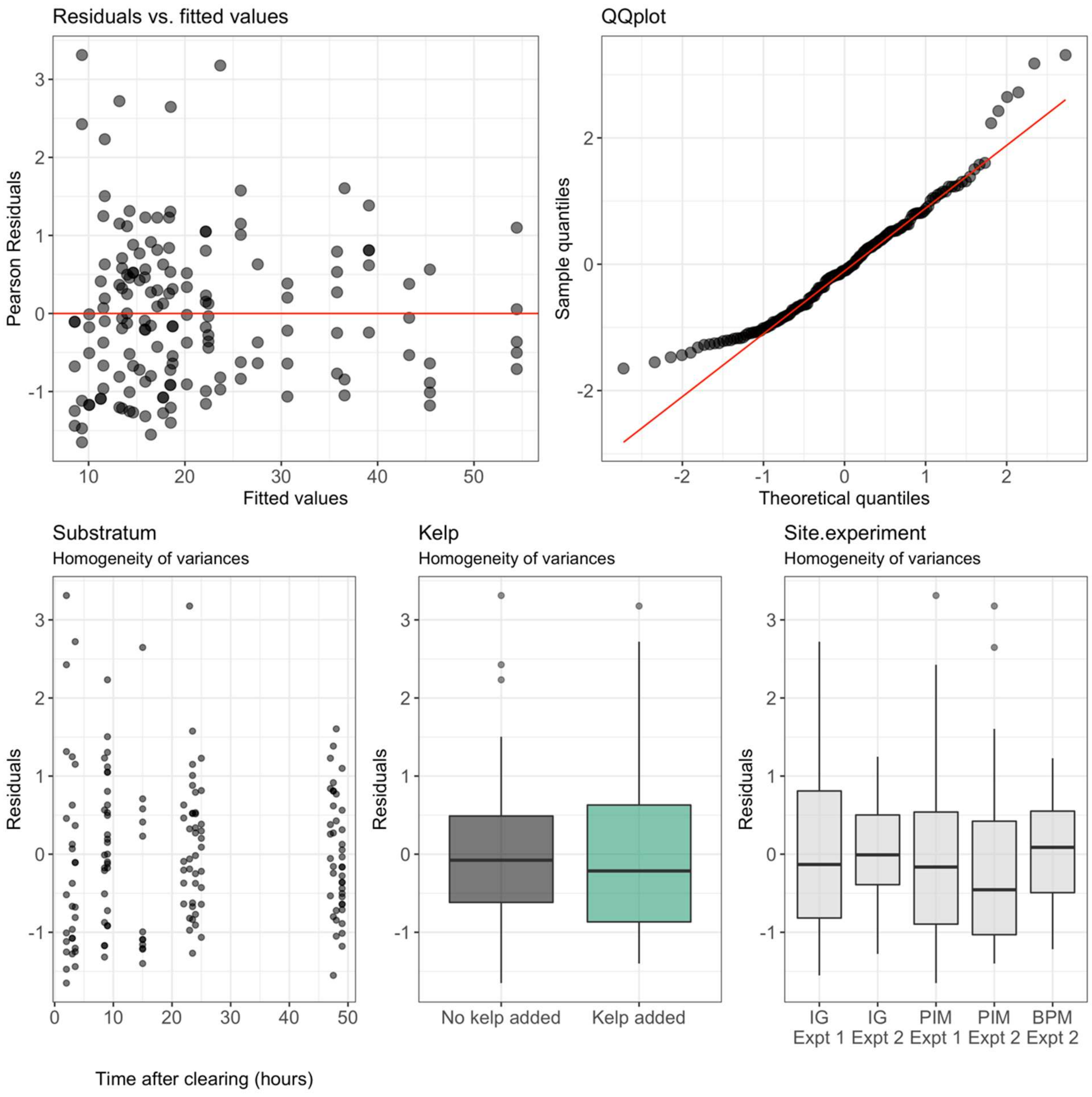

Figure S4. Visual evaluation of model fit and assumptions for the inside zone. The top two panels show residuals versus fitted values with no worrying patterns in the spread (left) and a quantile-quantile plot showing not terrible fit; with a negative binomial distribution, this evaluation (of normality) is much less important. The bottom three plots show residuals by the

three factors included in the model (Time after clearing, Kelp, and Site.exp) with no extreme heterogeneity of residuals evident.

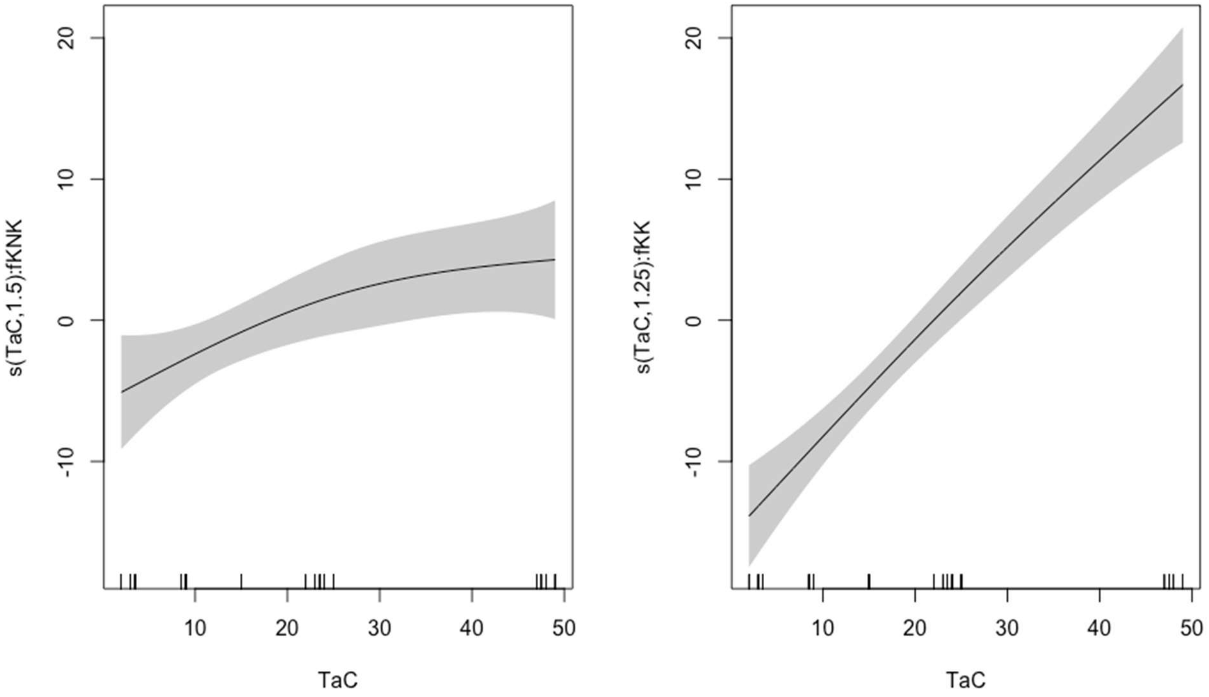

Figure S5. Smoothing splines showing the patterns through time of the no-kelp-added treatment (left panel) and the kelp-added treatment (right panel).

Outside zone

Model fit was verified visually using residual plots and qqplots (GLM portion of the model; Fig. S6) and plots of estimated splines (Fig. S7).

```
> summary(M3.out)

Family: Negative Binomial(10.652)
Link function: log

Formula:
Den ~ fSet_Site + fK + s(TaC, by = fK)
```

```
Parametric coefficients:
                 Estimate Std. Error z value Pr(>|z|)
(Intercept)      2.854062   0.097442  29.290   <2e-16 ***
fSet_SiteIG_9    0.262147   0.110745   2.367   0.0179 *
fSet_SitePIM_4 -0.142566   0.129120  -1.104   0.2695
fSet_SitePIM_8 -0.049503   0.112173  -0.441   0.6590
fSet_SitePM_7  -0.005093   0.111704  -0.046   0.9636
fKK              0.031234   0.061886   0.505   0.6138
---
Signif. codes:  0 '***' 0.001 '**' 0.01 '*' 0.05 '.' 0.1 ' ' 1

Approximate significance of smooth terms:
              edf Ref.df Chi.sq  p-value
s(TaC):fKNK 1.981  2.311  22.98 2.21e-05 ***
s(TaC):fKK  1.973  2.303   8.28   0.0306 *
---
Signif. codes:  0 '***' 0.001 '**' 0.01 '*' 0.05 '.' 0.1 ' ' 1

R-sq.(adj) =  0.208   Deviance explained = 24.7%
-REML = 534.75  Scale est. = 1         n = 156
```

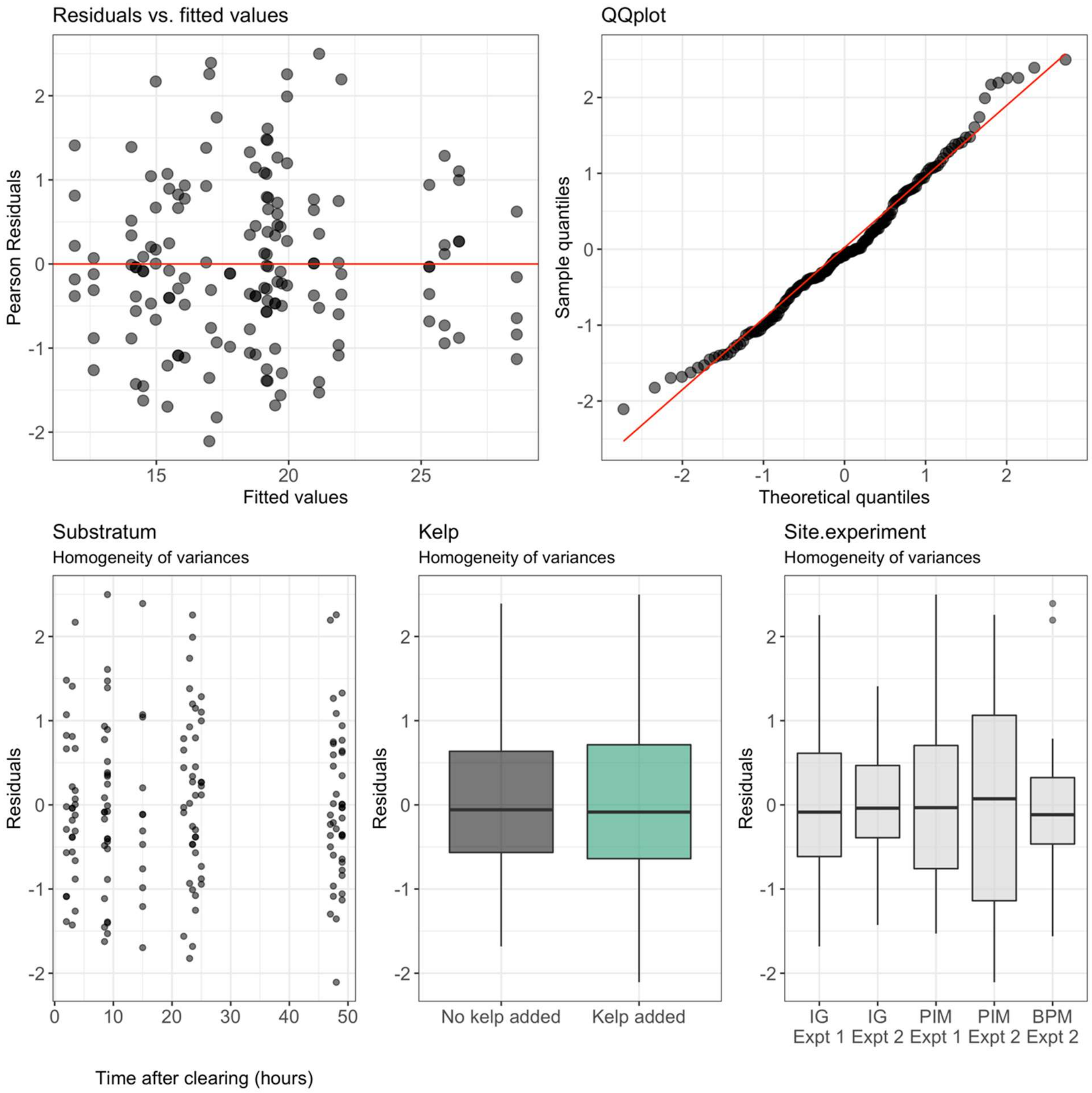

Figure S6. Visual evaluation of model fit and assumptions for the outside zone. The top two panels show residuals versus fitted values with no worrying patterns in the spread (left) and a quantile-quantile plot showing good fit; with a negative binomial distribution, this evaluation (of normality) is much less important. The bottom three plots show residuals by the three factors included in the model (Time after clearing, Kelp, and Site.exp) with no extreme heterogeneity of residuals evident.

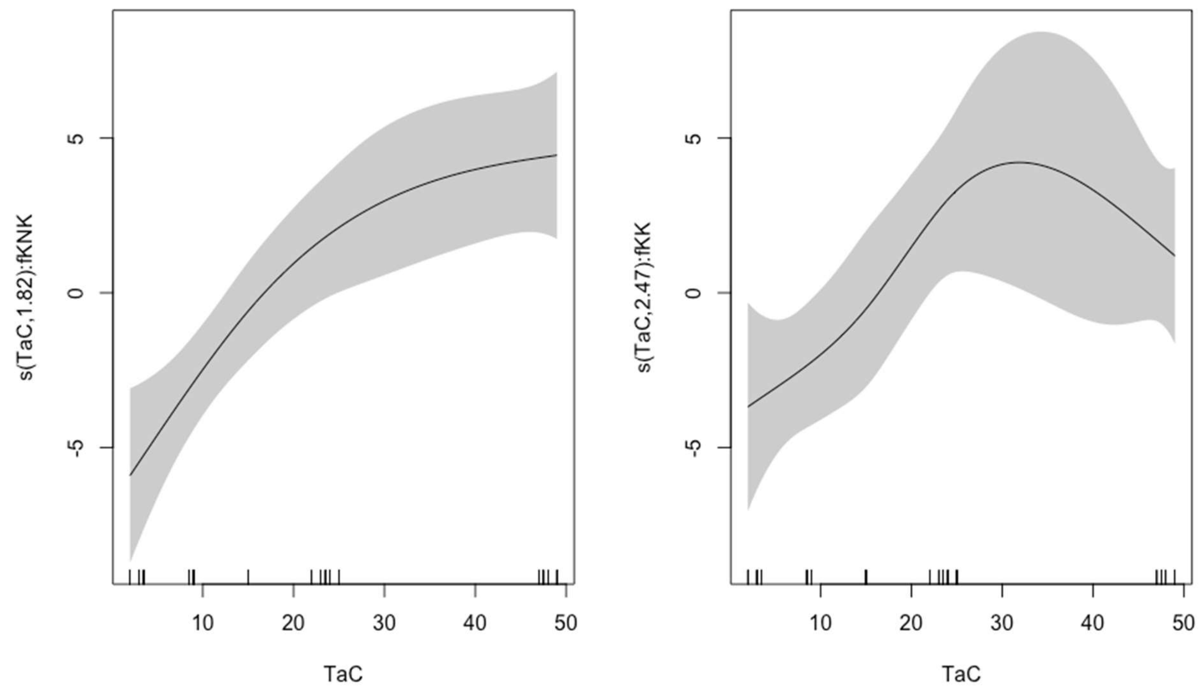

Figure S7. Smoothing splines showing the pattern through time of the no-kelp-added treatment (left panel) and the kelp-added treatment (right panel).

4. Consumption of drift kelp: experiments 1 and 2

To describe how consumption of drift kelp was affected by the numbers of urchins actively grazing and the substratum barriers installed, all kelp consumption data from the 24- and 48-hr visits from both experiments (barrier experiment and attraction/retention experiment) were analyzed together. We used the beta function from the betareg package (Zeileis et al., 2021) and then visually evaluated model fit (Fig. S8) and calculated variance inflation factors.

$$Proportion \sim Site.exp + \ Urchins\ on\ kelp + Substratum + Urchins{:}Substratum$$

Model formula in R notation:

```
> form <- prop_consum ~ fSet_Site + Urchins + fSub + Urchins:fSub
```

fSet_Site = Site.experiment
Urchins = Urchins touching kelp
fK = Kelp
fSub = Substratum treatment

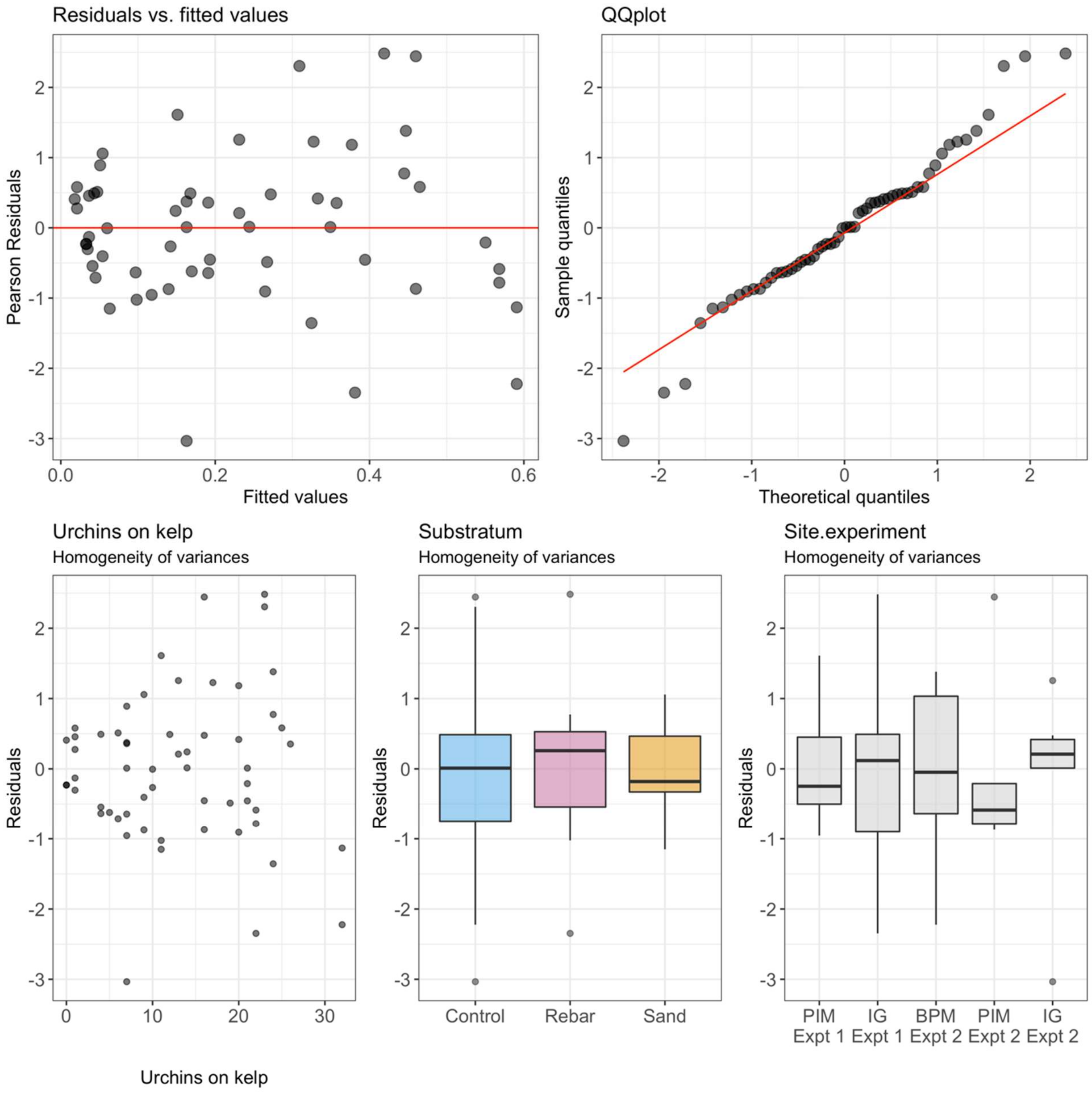

Figure S8. Visual evaluation of model fit and assumptions. The top two panels show residuals versus fitted values with no worrying patterns in the spread (left) and a quantile-quantile plot showing good fit. The bottom three plots show residuals by the three factors included in the model with no extreme heterogeneity of residuals evident, although there is more difference here between Site.experiment levels treatments than is ideal.

Variance inflation factors (see below) are low ($GVIF^{\wedge(1/(2*Df)} > 4$) in all cases, indicating no problems with the model.

```
                    GVIF Df GVIF^(1/(2*Df))
```

```
fSet_Site      2.480303  4         1.120245
Urchins        2.241469  1         1.497154
fSub          17.272581  2         2.038634
Urchins:fSub  12.473585  2         1.879307
```

We then tested whether the mean proportion of kelp consumed (Control replicates only) across both experiments could be explained by relative water movement (clod card loss). Replicates of kelp consumption were averaged within each 24-hour period of each experimental deployment to give a single estimate of consumption per 24-hour period which was matched with 24-hour relative water movement for the analysis. We again fit a beta regression (bounded proportional data) using the beta function from the betareg package and visually evaluated model fit (Fig. S9).

$$Mean\ proportion\ consumed \sim Relative\ water\ movement$$

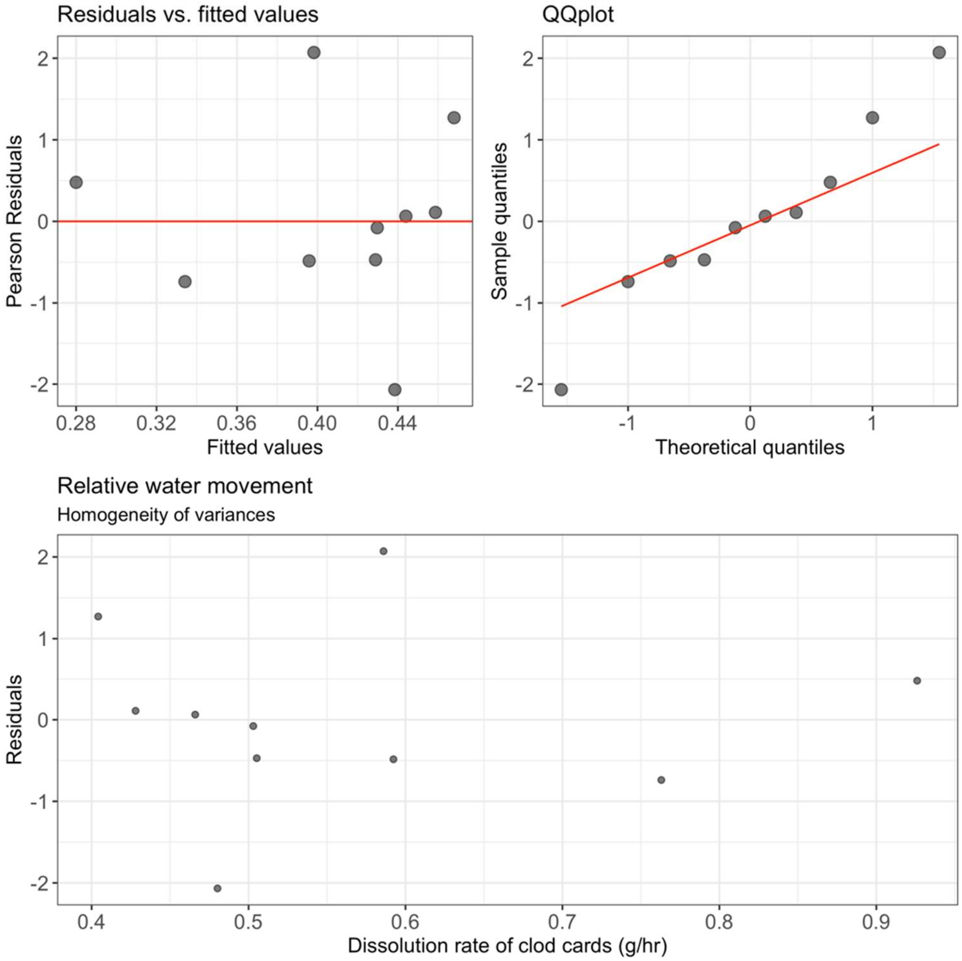

Figure S9. Visual evaluation of model fit and assumptions. The top two panels show residuals versus fitted values with no worrying patterns in the spread (left) and a quantile-quantile plot showing good fit; with so few points, patterns could be difficult to identify, however. The bottom plot shows residuals by dissolution rate with no extreme heterogeneity of residuals evident. Again, with so few points, however, patterns could be hard to detect.

References

Ripley, B., Venables, B., Bates, D. M., Hornik, K., Gebhardt, A., & Firth, D. (2022). *Support Functions and Datasets for Venables and Ripley's MASS*. Retrieved from http://www.stats.ox.ac.uk/pub/MASS4/ Contact

Wood, S. (2022). *mgcv: Mixed GAM computation vehicle with automatic smoothness estimation*. doi: 10.1201/9781315370279

Zeileis, A., Cribari-Neto, F., Gruen, B., Kosmidis, I., Simas, A. B., & Rocha, A. V. (2021). *betareg: Beta Regression*.
